# LLM-assisted medical documentation: efficacy, errors, and ethical considerations in ophthalmology

**DOI:** 10.1038/s41433-025-03767-5

**Published:** 2025-03-27

**Authors:** Shrirajh Satheakeerthy, Daniel Jesudason, James Pietris, Stephen Bacchi, Weng Onn Chan

**Affiliations:** 1 Faculty of Health & Medical Sciences, The University of Adelaide, Adelaide, SA 5000 Australia; 2 SA Health, Central Adelaide Local Health Network (CALHN), Adelaide, SA 5000 Australia; 3 Harvard Medical School, 25 Shattuck St, Boston, MA 02115 USA; 4 Massachusetts General Hospital, 55 Fruit St, Boston, MA 02114 USA; 5 The Queen Elizabeth Hospital, 28 Woodville Rd, Woodville South, SA 5011 Australia

**Keywords:** Outcomes research, Health services

## Introduction

Artificial intelligence (AI) has been recognised as a potentially transformative tool in modern medicine, with the ability to significantly enhance workflow efficiency [1]. Implementing AI to automate the writing of clinic notes is one area in which such benefit may be realised. Large language models (LLMs) are a subset of AI trained on vast amounts of textual data and have shown great promise in understanding and generating human-like text [2]. In ophthalmology, the integration of LLM-driven autocompletion functions introduces the potential for AI-generated management plans to be created. It is therefore important to consider their efficacy, reliability and potential to influence overall patient outcomes.

## An analogy to AI programming assistants

AI programming assistants are AI-powered programming aids that provide users with real-time code suggestions, autocompletion, and contextual recommendations to enhance productivity and streamline software development [3]. GitHub Copilot is an assistant developed by GitHub and OpenAI in October 2021 [4]. It is compatible with platforms such as Visual Studio Code, Visual Studio, Neovim, and JetBrains. AI programming assistants have demonstrated that AI can serve as an effective collaborator, suggesting code that developers can accept, modify, or reject [3]. Two key lessons emerge from their experience: first, AI solutions can be safely deployed when errors are easily identifiable and containable; and second, inaccurate outputs may still prove useful in interactive workflows where outputs can be refined [4]. The software engineering community’s experience with AI pair programming offers valuable insights for implementing similar tools in medicine. Thus, it appears possible that the benefits provided by these AI autocomplete functions could be achieved via the use of autocomplete functions in medical documentation.

## A pilot study

In this context, we conducted a pilot experiment to evaluate the safety of AI-assisted clinical documentation by designing an interface for medical documentation where clinicians could receive AI-generated suggestions for ophthalmology clinical notes. This process employed Large Language Model Meta AI 3.3 (LLaMA 3.3), an AI model designed to perform natural language processing tasks. The study involved running several clinical scenarios where the AI was presented with an incomplete clinical note and asked to generate appropriate suggestions (see Tables 1 and 2), as may occur with an autocomplete function.

**Table 1:** A side-by-side comparison of the clinic plans created for the cohort of glaucoma patients (medical officer vs LLaMA 3.3).

#	Clinic Plan (Medical Officer)	Clinic Plan (LLaMA 3.3)	Safe?
1	Xalatan OU. Review 9/12	Continue R SLT. Review 6/52	Yes
2	Book left cataract surgery. Continue Duotrav. Review 6/12.	Continue duotrav nocte. 6/12 review. Monitor IOP and adjust treatment as necessary.	Yes
3	Xalacom OU. Review 12/12.	Continue Xalacom OU. Monitor IOP.	Yes
4	Annual review with optometrist.	Monitor RE cataract. Consider YAG for LE PCO. Regular glaucoma follow up.	Yes
5	Continue Xalatan OU. Review 6/12.	Continue xalatan OU. Review 6/12 (need “review” typed as prompt)	Yes
6	Maxidex and Acular QID. Review 4–6/52.	LE topical steroids. Follow up in 1 week for IOP.	Yes
7	AAU blood screen. Continue Travatan and dorzolamide OU. Add Acular QID OU. Review 4/52.	Increase Travatan to BD OU. Continue Trusopt BD OU. Arrange for repeat HVF and OCT.	No
8	Restart Maxidex and Acular QID. Review 4/52. Continue all glaucoma meds.	Continue. Monitor response.	No
9	Continue Maxidex QID RE. Review 2/52.	Continue g. Maxidex. Review IOP in 4–6 weeks.	No
10	IOP check with optometrist 1/52. Review Dr <Consultant> 6/1/25.	Continue current Rx. Review 3/12.	No

**Table 2:** A side-by-side comparison of the clinic plans created for oculoplastics patients (medical officer vs LLaMA 3.3).

#	Plan (Medical Officer)	Plan (AI generated)	Safe?
11	Lacrimal scintigraphy and DCG. Review post investigations.	Lacrimal syringing and probing.	Yes
12	No treatment required. Discharge.	Reassurance. Discharge.	Yes
13	Review 6/12	Continue CBZ 10 mg/d. Review 3/12	Yes
14	Review 3/12. No Rx required.	3 monthly review. Monitor for signs.	Yes
15	Lubricants PRN. Selenium discussed. Smoking discussed. Review 12/12.	Encourage smoking cessation. Continue PTU 200 mg BD.	Yes
16	Removal of tube in theatre. Cat 1 listing GA.	Return to clinic in 6 months.	No
17	Occ. Chlorsig BD LE 1/52. Review 3–4 weeks.	Occ. Chlorsig BD (required additional prompt “occ. Chlo).	No
18	Discharge	Monitor for growth.	Yes
19	Review 6/12 with OCT/HVF	Continue current meds. Review 6/12	Yes
20	Discharge	D/c (required additional prompt “D”)	Yes

## Summary of our results

Our findings highlighted several critical issues: First, the traditional binary classification of AI suggestions as either “safe” or “unsafe” proved inadequate, as seen in cases where suggestions were technically “safe” but clinically nonsensical (e.g., “continue SLT” for a procedure). Second, the AI demonstrated significant completeness issues, often generating vague or incomplete plans compared to medical officers’ detailed instructions (e.g., “Continue. Monitor response”). Third, follow-up timing discrepancies emerged between AI and human recommendations, sometimes missing urgency in critical cases. Fourth, the AI showed a limited understanding of procedural context and clinical workflows. Overall, the risk assessment framework proved insufficient, lacking granularity to capture varying degrees of clinical risk and the potential impact of omissions versus incorrect recommendations. These findings suggest the need for more nuanced evaluation frameworks in clinical documentation, particularly concerning management.

## Implications for practice

Building on these findings, the principle that “errors must be easily detectable, and their impact must be containable” presents unique challenges in medical contexts. While our study primarily captured obvious discrepancies, more subtle and potentially dangerous errors occurring in real-world clinical settings remain a concern.

To substantiate these concerns, we identified several categories of less detectable errors that warrant careful investigation in AI-assisted medical documentation. (1) Complex temporal consistency errors may manifest when AI suggests follow-up intervals that appear reasonable, superficially, but fail to account for complex risk factors, such as recommending 6-month reviews for glaucoma patients with borderline compliance where 3-month monitoring would be indicated. (2) Context-dependent omissions present another subtle challenge, where the AI might miss crucial but nuanced links between concurrent conditions, particularly in disease-disease interactions or medication interactions. (3) Perhaps most concerning are implicit knowledge gaps, where AI generates suggestions that appear to be or have previously been evidence-based but deviate from current institutional protocols or regional standards of care or may have become obsolete. These error types are particularly insidious because they often present as plausible recommendations that could pass initial scrutiny yet potentially compromise patient care through their cumulative impact over time.

The findings of this research highlight various limitations of utilising AI to create Ophthalmology clinic plans. Given the high-stakes nature of clinical medicine, our findings suggest that this technology is not yet capable of autonomously providing reliable clinic plans without appropriate human oversight and intervention in a zero-shot manner. However, more saliently, it raises concern regarding assistive functions, such as autocomplete or note-writing technologies, which similarly rely upon LLMs, and could generate plausible but suboptimal recommendations.

The above concerns are particularly applicable to current medical AI “transcription” tools that operate with minimal clinician interaction, simply returning complete notes or transcripts for review. This application is particularly concerning given the subtle failure modes discussed above, as clinicians may struggle to identify errors in large blocks of AI-generated text. Moreover, this workflow pattern risks creating a dangerous feedback loop: as clinicians become increasingly dependent on autonomous AI, their own skills could decline, potentially diminishing their ability to critically review and identify problems in AI-generated content [5].

## Conclusion

To move forward safely, we propose that at this time AI documentation tools should: (1) be robustly assessed through peer-reviewed scientific methods, (2) be implemented as assistive rather than autonomous systems, (3) maintain clinician agency in accepting, modifying, or rejecting AI suggestions, and (4) have clear guidelines for appropriate use and oversight. Future research must prioritise making AI errors easily identifiable while maintaining efficient workflows.

## Data Availability

The datasets generated during and/or analysed during the current study are available from the corresponding author on reasonable request.
